# Transcriptional responses of skeletal stem/progenitor cells to hindlimb unloading and recovery correlate with localized but not systemic multi-systems impacts

**DOI:** 10.1038/s41526-021-00178-0

**Published:** 2021-11-26

**Authors:** Cori N. Booker, Christopher L. Haga, Siddaraju V. Boregowda, Jacqueline Strivelli, Donald G. Phinney

**Affiliations:** Department of Molecular Medicine, The Scripps Research Institute – Scripps Florida, Jupiter, Florida 33458 USA

**Keywords:** Stem cells, Systems biology

## Abstract

Disuse osteoporosis (DO) results from mechanical unloading of weight-bearing bones and causes structural changes that compromise skeletal integrity, leading to increased fracture risk. Although bone loss in DO results from imbalances in osteoblast vs. osteoclast activity, its effects on skeletal stem/progenitor cells (SSCs) is indeterminate. We modeled DO in mice by 8 and 14 weeks of hindlimb unloading (HU) or 8 weeks of unloading followed by 8 weeks of recovery (HUR) and monitored impacts on animal physiology and behavior, metabolism, marrow adipose tissue (MAT) volume, bone density and micro-architecture, and bone marrow (BM) leptin and tyrosine hydroxylase (TH) protein expression, and correlated multi-systems impacts of HU and HUR with the transcript profiles of Lin^−^LEPR^+^ SSCs and mesenchymal stem cells (MSCs) purified from BM. Using this integrative approach, we demonstrate that prolonged HU induces muscle atrophy, progressive bone loss, and MAT accumulation that paralleled increases in BM but not systemic leptin levels, which remained low in lipodystrophic HU mice. HU also induced SSC quiescence and downregulated bone anabolic and neurogenic pathways, which paralleled increases in BM TH expression, but had minimal impacts on MSCs, indicating a lack of HU memory in culture-expanded populations. Although most impacts of HU were reversed by HUR, trabecular micro-architecture remained compromised and time-resolved changes in the SSC transcriptome identified various signaling pathways implicated in bone formation that were unresponsive to HUR. These findings indicate that HU-induced alterations to the SSC transcriptome that persist after reloading may contribute to poor bone recovery.

## Introduction

Osteoporosis is characterized by low bone mass and deleterious alterations to bone micro-architecture that increase fracture risk and decrease fracture healing^1,2^. Although aging is a leading cause of osteoporosis, prolonged periods of disuse that occur with therapeutic bed rest, paralysis, and limb amputation also result in osteoporosis of weight-bearing bones due to mechanical unloading, regardless of age^1,3,4^. During spaceflight, an extreme form of disuse, bone loss is accelerated and recovery after return to normal gravity is slow and incomplete^3^. Due to the poor adaptation of the skeleton to mechanical unloading, disuse osteoporosis (DO) results in long-term complication for patients and represents a major obstacle to manned space exploration.

Bone loss in DO is largely attributed to imbalances in bone resorption vs. accrual, which is tightly regulated by the coordinated actions of mineralizing osteoblasts, resorptive osteoclasts, and mechano-sensing osteocytes^5–10^. Bone remodeling is also regulated locally by autocrine and paracrine signaling, and systemically via the action of hormones, cytokines, and neurotransmitters^11,12^. Accordingly, anti-resorptive bisphosphonates^13,14^, selective estrogen receptor modulators^15^, osteoprotegrin^16,17^, and β-adrenergic agonists^18–21^ mitigate bone loss to varying degrees in rodent DO models by targeting these processes. Recent studies have identified several putative skeletal stem/progenitor cell (SSC) populations that function as precursors of bone and fat tissue in adult bone marrow (BM)^22–25^, and emerging data indicate that altered SSC function in response to disease also contributes to skeletal pathology. For example, increased adipogenesis of LEPR^+^ SSCs in response to systemic leptin signaling was linked to increased marrow adipose tissue (MAT) accumulation in a mouse model of diet-induced obesity^26^, and LEPR^+^ SSCs were also shown to represent the main source of fibrogenic cells in primary myelofibrosis^27^. Nevertheless, few studies have directly examined impacts of HU and recovery on SSCs isolated prospectively from marrow. Rather, impacts have been inferred from cell-based studies of mesenchymal stem/stromal cells (MSCs) cultured under simulated microgravity^28–34^, or by analysis of unfractionated or plastic-adherent BM cells recovered from mice subject to hindlimb unloading (HU) or spaceflight^35,36^. To address these gaps, we modeled DO and recovery in adult mice using a modifed version of the Morey–Holton HU technique^37^ and quantified impacts on mouse physiology, behavior, metabolism, skeletal pathology, marrow adiposity, and BM innervation. We also used RNA-sequencing (RNA-seq)-based transcript profiling to delineate impacts of HU and HU recovery (HUR) on Lin^−^LEPR^+^ SSCs and MSCs enriched from BM to high purity. This intergrative approach provides a comprehensive assessment of multi-systems impacts of HU in mice, resolves differences in the sensitivity of SSCs and culture-expanded MSCs in this model, and also evaluates impacts of an equivalent period of reloading.

## Results

### HU induces localized skeletal pathology and muscle atrophy

**Fig. 1:**
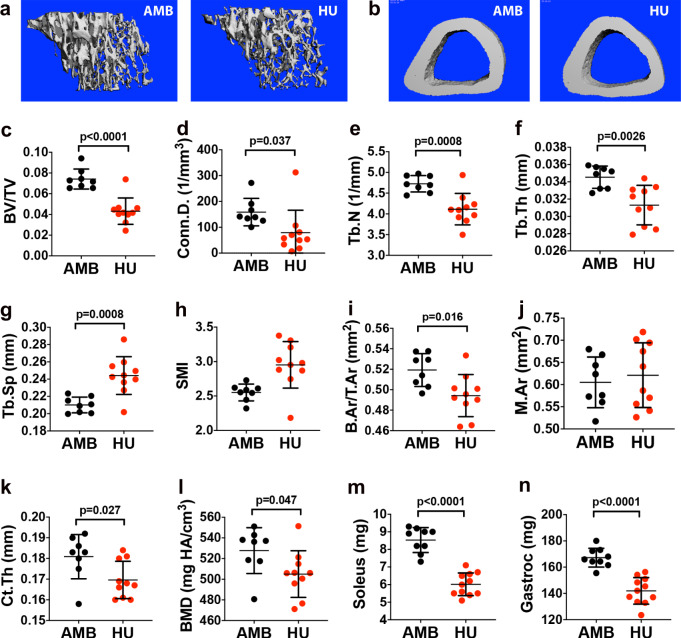
Mice subjected to 8wk of HU exhibit localized skeletal pathology and muscle atrophy. **a**, **b** Representative micro-CT images of the proximal (**a**) and distal tibia (**b**) from 8wk HU mice and their respective AMB controls. **c**–**h** Quantification of BV/TV (**c**), Conn.D. (**d**), Tb.N. (**e**), Tb.Th. (**f**), Tb.Sp. (**g**), and SMI (**h**) by micro-CT analysis (*n* = 8–10 mice/group). **i**–**l** Quantification of B.Ar/T.Ar (**i**), M.Ar. (**j**), Ct.Th. (**k**), and BMD (**l**) by micro-CT analysis (*n* = 8–10 mice/group). **m**, **n** Quantification of soleus (**m**) and gastrocnemius (**n**) wet mass (*n* = 9–14 mice/group). Data represent mean ± SD. All *p*-values are by Student’s *t*-test (AMB vs. HU mice).

To confirm that 8wk of continuous HU-induced skeletal pathology, long bone morphology was evaluated by micro-computed tomography (CT). Analysis of proximal tibiae (Fig. 1a) revealed significant decreases in trabecular bone volume as a fraction of total volume (BV/TV) (Fig. 1c), trabecular connectivity density (Conn.D) (Fig. 1d), trabecular number (Tb.N) (Fig. 1e), trabecular thickness (Tb.Th) (Fig. 1f), and significant increases in trabecular spacing (Tb.Sp) (Fig. 1g) in HU mice as compared to age- and sex-matched ambulatory controls (AMB). Structure model index (SMI) also trended upward in HU vs. AMB mice (Fig. 1h), indicating trabecular bone loss resulted from thinning of plate-like to rod-like structures^38^. Analysis of tibiae midshafts (Fig. 1b) further showed that HU significantly decreased cortical bone area as a fraction of total area (B.Ar/T.Ar) (Fig. 1i), cortical thickness (Ct.Th) (Fig. 1k) and bone mineral density (BMD) (Fig. 1l) without affecting total marrow area (Fig. 1j). As expected based on skeletal impacts, HU mice also exhibited significant atrophy of the soleus (Fig. 1m) and gastrocnemius (Fig. 1n) muscles. Therefore, these impacts of HU are consistent with a phenotype of DO.

### HU-induced MAT accumulation correlates with localized increases in BM leptin and TH expression

**Fig. 2:**
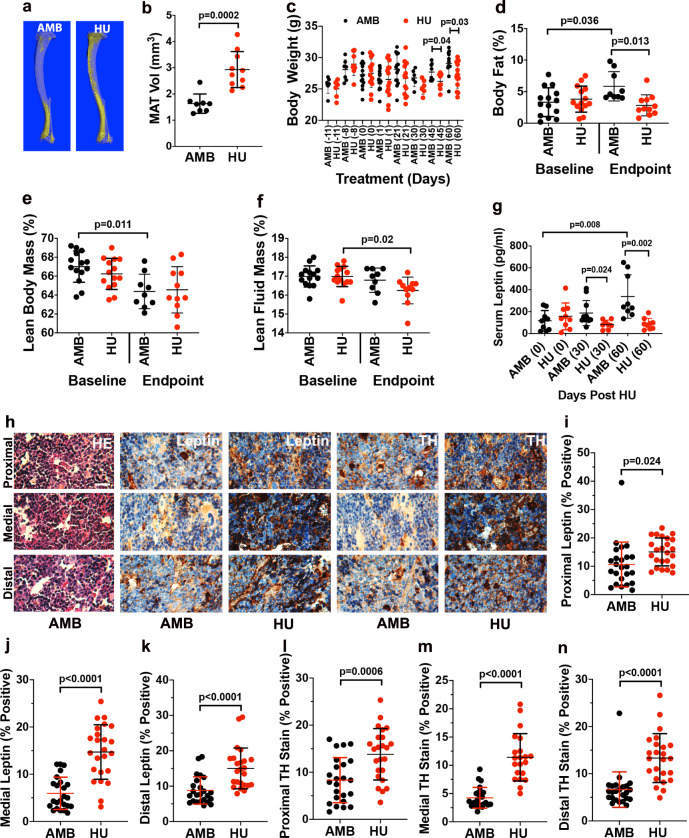
HU induces lipodystrophy, MAT accumulation, and increased BM leptin and TH expression. **a**, **b** Representative micro-CT images of osmium tetroxide-stained tibiae from 8wk HU and AMB mice (**a**) and corresponding volumetric measurements of MAT (**b**) (*n* = 8–10 mice/group). **c** Time course of weight gain in 8wk HU and AMB mice wherein numbers in parenthesis are days post-HU, which began at day 1 (*n* = 6–14 mice/group). **d**–**f** NMR quantification of total body fat (**d**), lean body mass (**e**), and lean fluid mass (**f**) (*n* = 9–14 mice/group). **g** Endpoint measures of serum leptin levels in 8wk HU and AMB mice (*n* = 8–10 mice/group). **h** Representative photomicrographs of femoral bone tissue sections from 8wk HU and AMB mice stained with HE or antibodies against leptin or TH (×100, scale bar = 50 μm). **i**–**n** Semi-quantitative analysis of leptin levels in the proximal (**i**), medial (**j**), and distal femur (**k**), and TH levels (**l**–**n**) in the proximal (**l**), medial (**m**), and distal femur (**n**) (*n* = 2 mice/group). Data represent mean ± SD. *p*-Values in **b**, **c**, **i**–**n** are by Student’s *t*-test (AMB vs. HU mice at each time point) and in **d**–**g** by one-way ANOVA with Tukey’s post hoc test.

Micro-CT analysis of tibiae (Fig. 2a) also demonstrated that 8wk of HU-induced significant increases in MAT volume (Fig. 2b). However, monitoring of body weight showed that HU mice weighed significantly less after 45d and 60d of HU as compared to their corresponding AMB controls (Fig. 2c) and whole-body NMR confirmed that AMB but not HU mice exhibited significant increases in endpoint vs. baseline measures of body fat mass (Fig. 2d) and significant decreases in lean body mass (Fig. 2e), whereas HU mice exhibited a slight but significant decrease in fluid mass (Fig. 2f). Consistent with these results, enzyme-linked immunosorent assay (ELISA) confirmed that mid and endpoint measures of serum leptin were significantly lower in HU vs. AMB mice (Fig. 2g), whereas blood panels confirmed that all mice were healthy and free of metabolic disease throughout the study time course (Supplementary Fig. S1). Together, these results indicate that 8wk of HU induces significant lipodystrophy in mice, and as such serum leptin levels do not correlate with MAT accumulation as observed in diet-induced obese mice^26^. To determine whether HU induces localized changes in BM, we analyzed tissue sections by immuno-histochemical staining (Fig. 2h), which revealed significant increases in BM leptin expression throughout the proximal, medial, and distal femur in HU vs. AMB mice (Fig. 2i–k). These incongruent compartmental impacts of HU on leptin expression indicate BM adiposity correlates with BM but not serum leptin levels. As mouse and human MAT is capable of expressing leptin^39,40^, which acts directly on SSCs to inhibit bone formation^26^, these data further suggest that BM leptin signaling may play a more dominant role in disuse-related bone loss than previously appreciated. Increased sympathetic signaling is also linked to bone loss through increased osteoclastogenic activity and bone catabolism^41,42^, and immunohistochemistry (IHC) staining also revealed elevated expression of tyrosine hydroxylase (TH) in the femurs of HU vs. AMB mice (Fig. 2h, l–n). As TH is a marker of sympathetic never fibers in BM^43,44^ and mice lacking expression of the β_2_-adrenergic receptor exhibit a high bone mass phenotype^45^, these data suggest that HU-induced increases in local sympathetic innervation also contribute to bone loss in this model.

### HU mice exhibit elevated corticosterone levels and increased activity but normal circadian rhythms

HU is known to represent a significant stressor and elevate plasma glucocorticoid levels in mice^46^. Consistent with this fact, blood profiling revealed that HU mice exhibited elevated serum corticosterone levels for up to 28 days post-HU (Supplementary Fig. S2a). Behavioral analysis also revealed that AMB mice moved a greater total distance during the first days of observation (Supplementary Fig. S2b), which reflects unrestricted access to the novel cage environment followed by habituation, but thereafter no significant difference in distance moved was noted between treatment groups. Using a more sensitive static activity metric, HU mice were shown to be more active each day and spent a significantly greater percentage of time each day being active (Supplementary Fig. S2c, d) but no difference in diurnal and nocturnal behavior patterns was noted between experimental groups (Supplementary Fig. S2e). Together, these data indicate that HU did not disrupt circadian cycling but increased activity, which is consistent with stress-associated repetitive behaviors observed in rats undergoing HU^47^ and in space flown mice^48^. Increased activity may account for the failure of HU mice to gain body fat and underscores the importance of stress hormone-mediated behavioral adaptations to HU on physiology. Elevated stress hormone levels are also associated with sympathetic nerve overgrowth^49,50^, which provides a potential mechanism for observed increases in TH expression in the BM of HU mice. Importantly, in these studies mice were maintained at 21 °C and not under thermo-neutral housing conditions. Therefore, although AMB control mice were free to participate in normal nesting behavior to regulate body temperature, mice in the HU position were not able to do so. Therefore, elevated caloric burning to maintain body temperature may also have contributed to the failure to gain body weight and body fat mass observed in HU vs. AMB mice.

### HU has disparate effects on SSC and MSC gene expression and promotes SSC quiescence

**Fig. 3:**
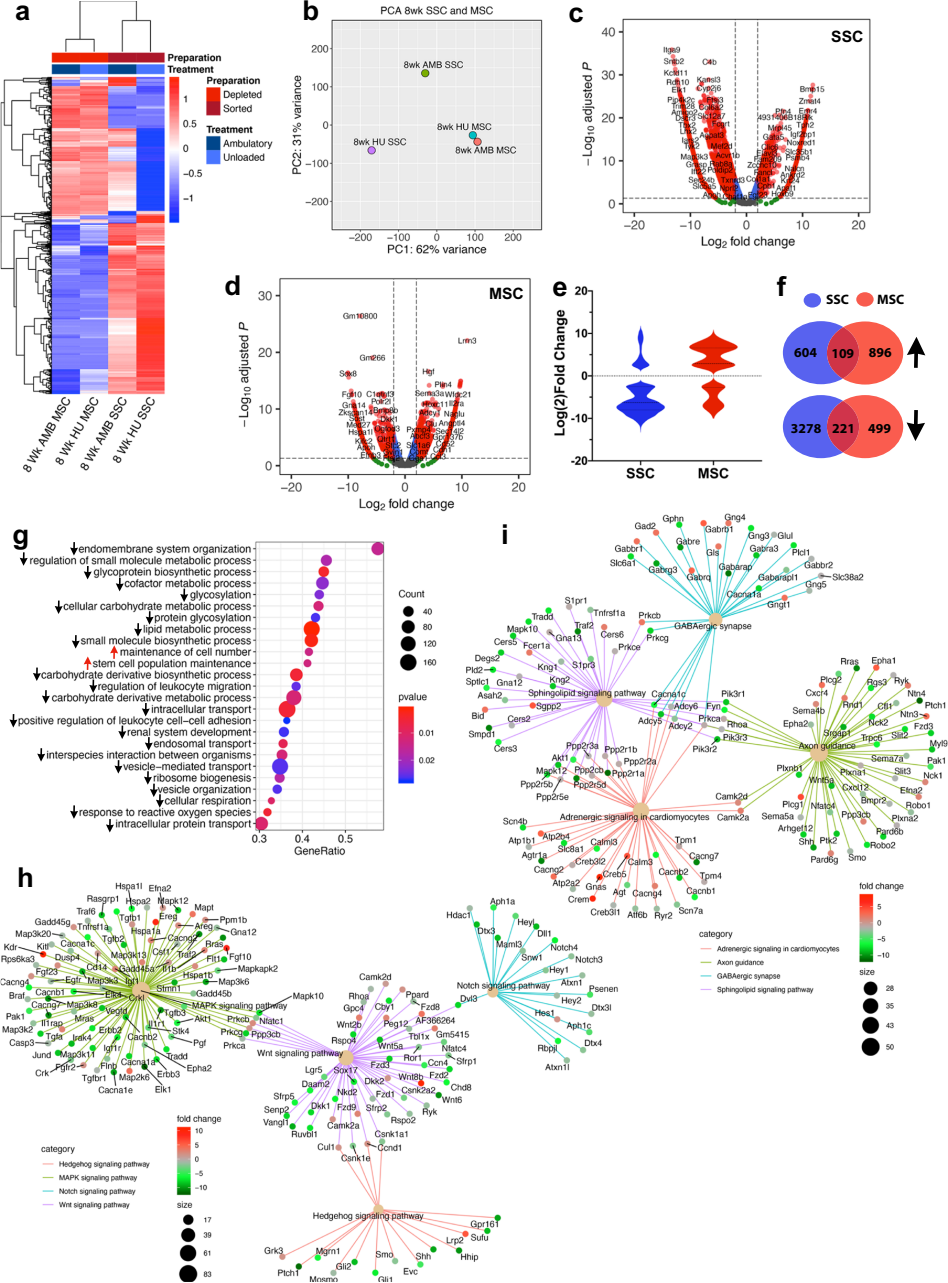
Transcriptional profiling reveals disparate impacts of 8wk HU on SSCs and MSCs. **a** Heat map of the top 1000 DEGs in SSCs and MSCs from 8wk HU vs. AMB mice as determined by RNA-seq analysis. Colors correspond to per-gene *z*-score computed across each row. **b** PCA analysis of data from **a**. **c**, **d** Volcano plots showing Log2 fold-change (FC) values for DEGs and their corresponding *p*-values in SSCs and MSCs from 8wk HU vs. AMB mice. Red circles denote DEGs with Log2 FC > 2. **e** Violin plot of data from (**c**, **d**) showing number of DEGs up or downregulated by >Log2 fold in SSCs or MSCs in response to HU. **f** Venn diagram showing overlap of upregulated (109/1718, 6%) and downregulated (211/3777, 15.5%) DEGs common to SSCs and MSCs. **g** Top GO terms based on p-value identified by GSEA of DEGs in SSCs from 8wk HU vs. AMB mice. Red arrows indicate upregulated and black arrows indicate downregulated gene sets corresponding to each GO term. **h**, **i** KEGG pathway analysis showing fold-change of DEGs within gene regulatory networks related to environmental information processing (**h**) and organismal systems (**i**).

Next, we isolated CD31^−^CD45^−^Ter119^−^LEPR^+^ (Lin^−^LEPR^+^) SSCs from pooled BM (*N* = 3 mice) of mice subjected to 8wk of HU and AMB controls using a modified protocol involving immuno-depletion and fluorescence-activated cell sorting (FACS), to ensure high purity (Supplementary Fig. S3a–d). As expected, Lin^−^LEPR^+^ SSCs retained all the CFU-F activity in BM but lacked hematopoietic colony-forming activity (Supplementary Fig. S3e, f). SSC yields from HU vs. AMB mice also trended lower but were not statistically significant (Supplementary Fig. S3g). As culture-adapted MSCs are widely used as surrogates for SSCs, we also enriched MSCs to high purity via immuno-depletion from BM pooled from a separate cohort of mice (*N* = 3 mice)^51^. SSCs and MSCs from HU mice and their respective AMB controls were then subjected to RNA-seq analysis. Hierarchical clustering of the top 1000 expressed transcripts segregated populations by isolation method (Fig. 3a) and principle-component analysis (PCA) revealed HU altered gene expression in SSCs to a greater extent than MSCs (Fig. 3b). Herein, PC1 genes mapped to Gene Ontology (GO) terms related to cytokine production, peptide secretion, and regulation of inflammatory and stress responses, whereas PC2 genes mapped to terms related to regulation of peptidase activity and proteolysis, response to corticosteroids, cell adhesion, and developmental processes (Supplementary Fig. S4a). This analysis also identified 3991 and 1504 differentially expressed genes (DEGs) in SSCs (Fig. 3c) and MSCs (Fig. 3d), respectively, which were significantly altered in HU. Most DEGs were downregulated in HU SSCs but upregulated in HU MSCs (Fig. 3e), and the up- and downregulated genes were highly discordant between cell types (Fig. 3f). Moreover, GO terms enriched in HU vs. AMB MSCs are characteristic of actively proliferating cells (Supplementary Fig. S4b) and as such reflect cell adaptation to tissue culture. These data suggest that immuno-depletion and culture expansion largely erase gene expresson patterns induced by HU in MSCs, thus indicating that direct isolation of SSCs is a preferable method for studying the effects of HU on stem/progenitor function.

Gene set enrichment analysis (GSEA) identified “maintenance of cell number” and “stem cell population maintenance” as the only GO terms significantly upregulated in SSCs following HU (Fig. 3g). DEGs in these highly related GO terms include chromatin-modifying enzymes, DNA repair enzymes, and developmentally regulated transcriptional activators. This result is consistent with reports showing that spaceflight induces stem cells to adopt a de-differentiated phenotype^52,53^. In contrast, GO terms related to metabolic/biosynthetic processes involving glycoproteins, carbohydrates, small molecules, lipids, cofactors and organophosphates, and biological processes related to cellular respiration, endosomal transport, vesicle organization, and ribosome biogenesis were all significantly downregulated (Fig. 3g). Kyoto Encyclopedia of Genes and Genomes (KEGG) pathway analysis also identified several signaling networks downregulated by HU in SSCs including one involving Hedgehog, mitogen-activated protein kinase (MAPK), Notch, and WNT signaling (Fig. 3h), and one related to O- and N-glycan biosynthesis, carbon metabolism, fatty acid metabolism, arginine and purine metabolism, and glycosaminoglycan and terpenoid biosynthesis (Supplementary Fig. S4c). As hedgehog^25,54^ and Wnt pathways^55^ are directly implicated in SSC osteogenesis and bone homeostasis and Notch signaling regulates the size of the SSC pool in BM^56,57^, these results demonstrate that HU has negative impacts on stem/progenitor cells that lie upstream of the osteoblast lineage. KEGG analysis also identified an organismal systems network mapping to various neural pathways that was downregulated by HU (Fig. 3i), which is consistent with IHC data demonstrating HU-induced sympathetic hypertrophy in BM.

### Prolonged (14wk) HU induces progressive bone loss and promotes SSC quiescence

**Fig. 4:**
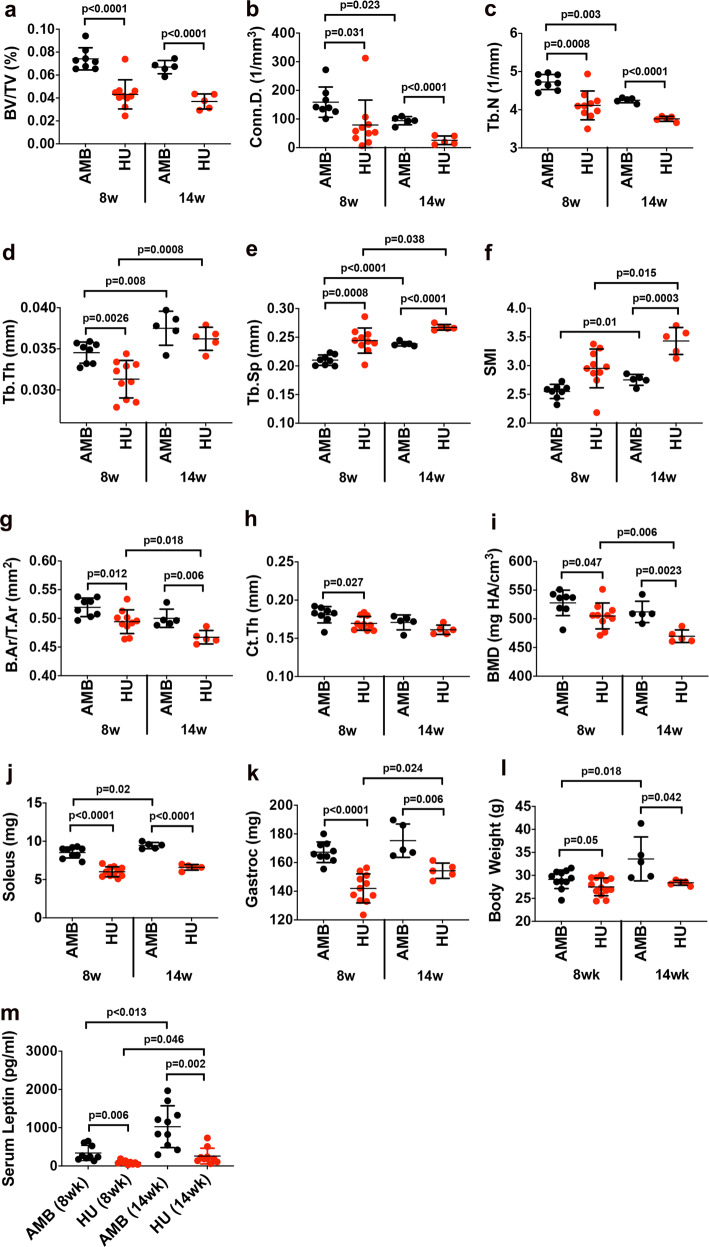
Prolonged HU induces progressive bone loss. **a**–**f** Quantification of BV/TV (**a**), Conn.D. (**b**), Tb.N. (**c**), Tb.Th. (**d**), Tb.Sp. (**e**) and SMI (**f**) by micro-CT analysis of the proximal tibiae from 8 and 14wk HU mice and their respective AMB controls (*n* = 5–10 mice/group). **g**–**i** Quantification of B.Ar/T.Ar (**g**), Ct.Th. (**h**), and BMD (**i**) by micro-CT analysis of the distal tibiae from mice in **a** (*n* = 5–10 mice/group). **j**–**m** Endpoint measures of soleus (**j**) and gastrocnemius (**k**) mass, body weight (**l**), and serum leptin levels (**m**) from 8 and 14wk HU mice and their AMB controls (*n* = 5–10 mice/group). Data are mean ± SD. *P*-values are by one-way ANOVA and Tukey’s post hoc test.

To contextualize the physiological impacts of prolonged disuse on skeletal pathology, we also subjected mice to 14wk of HU and compared outcomes to 8wk HU mice, which revealed that bone loss was progressive in this model. For example, while BV/TV (Fig. 4a), Conn.D (Fig. 4b), and Tb.N (Fig. 4c) were signficantly decreased in both HU cohorts compared to their respective AMB controls, these metrics also trended downward between 8wk and 14wk HU mice. Metrics of Tb.Th (Fig. 4d), Tb.Sp (Fig. 4e), and SMI (Fig. 4f) were also significantly different between HU vs. AMB mice after 8wk or 14wk of HU and between 8wk and 14wk HU mice, indicating that erosion of trabecular architecture was progressive in response to HU. Similar trends were also seen for measures of B.Ar/T.Ar (Fig. 4g) and BMD (Fig. 4i) but not Ct.Th (Fig. 4h), indicating that cortical bone loss was also progressive in this model. Many of these bone parameters also differed significantly between 14wk vs. 8wk AMB mice, which is consistent with studies showing that C57BL/6 mice reach peak bone density at 2 months of age and declines steadily thereafter^58^. In contrast, muscle atrophy (Fig. 4j, k) and weight loss (Fig. 4l) were not progressive in this model, whereas body weight (Fig. 4l) and serum leptin levels (Fig. 4m) increased significantly in 14wk vs. 8wk AMB mice as a result of normal aging.

We also performed RNA-seq analysis of SSCs and MSCs from 14wk AMB and HU mice, and hierarchical clustering and PCA analysis affirmed previous differences in cell sensitivity but identified a different subset of genes contributing to PC1 and PC2 (Supplementary Fig. 5a–c). Therefore, even 14wk of HU was not sufficient to imbue MSCs with a “memory” of HU. The total number of DEGs in SSCs and MSCs from 14wk HU vs. AMB mice were fewer than detected after 8wk of HU (Supplementary Fig. 5d, e) and these were predominantly downregulated and highly discordant between cell types (Supplementary Fig. 5f, g). GSEA of the SSC RNA-seq data identified GO terms “regulation of peptidase activity,” “negative regulation of catalytic activity,” and “membrane organization” as significantly upregulated, and “cell cycle,” “cell migration,” “cell adhesion,” and “reproductive processes” as significantly downregulated (Supplementary Fig. 5h). KEGG analysis identified molecular interaction pathways related to Parkinson’s, Alzheimer’s, Huntington’s, and non-alcoholic fatty liver disease as significantly altered in 14wk HU vs. AMB mice (Supplementary Fig. 5i), which are consistent with alterations in MAT and TH staining observed after 8wk of HU (Fig. 2), and again suggest a direct role of BM niche signaling in modulating SSC gene expression. By KEGG, “oxidative phosphorylation,” “thermogenesis,” “cell cycle,” “DNA replication,” “proteasome,” and “ribosome” were also significantly downregulated (Supplementary Fig. 5j), indicating that prolonged HU induced a deepening state of cellular quiescence in SSCs, thereby strengthening the association between DO-induced bone loss and SSC dysfunction.

### HUR restores muscle and body fat mass, BM leptin, and TH expression but not trabecular bone or SSC transcription

**Fig. 5:**
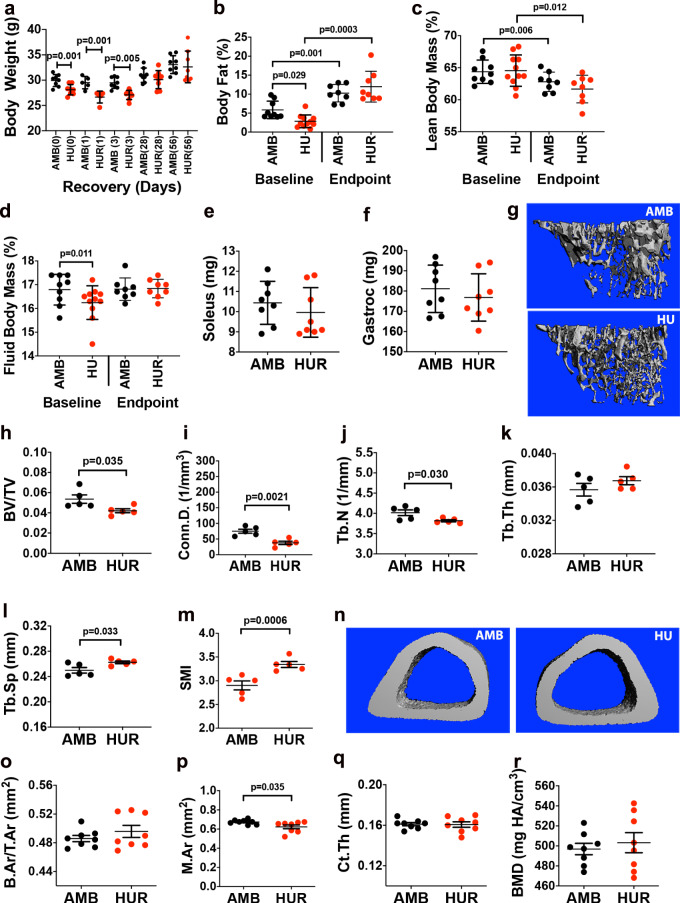
HUR reverses lipodystrophy, muscle atrophy, and cortical but not trabecular bone loss. **a** Time course of weight gain in HUR and AMB mice wherein numbers in parenthesis are days post-HUR, which began at day 1 (*n* = 6–8 mice/group). **b**–**d** NMR quantification of baseline and endpoint measures of body fat (**b**), lean (**c**), and fluid mass (**d**) of AMB and HUR mice (*n* = 8 mice/group). **e**, **f** Endpoint measures of soleus (**e**) and gastrocnemius (**f**) mass in AMB and HUR mice (*n* = 9–14 mice/group). **g**–**m** Representative micro-CT images of the proximal tibia (**g**) from AMB and HUR mice and quantification of BV/TV (**h**), Conn.D. (**i**), Tb.N. (**j**), Tb.Th. (**k**), Tb.Sp. (**l**), and SMI (**m**) (*n* = 8–10 mice/group). **n**–**r** Representative micro-CT images of the midshaft tibia (**n**) from AMB and HUR mice and quantification of B.Ar/T.Ar (**o**), M.Ar. (**p**), Ct.Th. (**q**), and BMD (**r**) (*n* = 8–10 mice/group). Data are mean ± SD. *P*-values in **a**, **e**, **f**, **h**–**m**, and **o**–**r** are by Student’s *t*-test (AMB vs. HU mice at each time point) and in **b**–**d** by one-way ANOVA with Tukey post hoc test.

Mice subject to 8wk HUR showed no significant difference in endpoint measures of body weight (Fig. 5a), body fat mass (Fig. 5b), lean mass (Fig. 5c), fluid mass (Fig. 5d), and muscle mass (Fig. 5e, f) as compared to age-matched AMB mice. However, micro-CT of the proximal tibiae (Fig. 5g) revealed small but significant differences in BV/TV (Fig. 5h), Conn. D (Fig. 5i), Tb.N (Fig. 5j), Tb. Sp (Fig. 5l), and SMI (Fig. 5m) but not Tb.Th (Fig. 5k) between AMB vs. HUR mice, indicating that reloading failed to induce full restoration of trabecular bone. Alternatively, analysis of midshaft tibiae (Fig. 5n) revealed no significant difference in B.Ar/T.Ar (Fig. 5o), Ct.Th (Fig. 5q), or BMD (Fig. 5r) between experimental groups, consistent with studies showing rapid restoration of cortical bone in response to weight-bearing activity^59,60^. Continuous monitoring of metabolic parameters (Supplementary Fig. 6a–d) revealed trends toward elevated blood glucose and TAG levels in both AMB and HUR mice, consistent with age-dependent increases in body weight and fat mass (Fig. 5a, b) but no significant differences in corticosterone levels (Supplementary Fig. 6e). IHC analysis of BM (Supplementary Fig. S7a) also showed no difference in leptin (Supplementary Fig. S7b–d) and TH (Supplementary Fig. S7e–g) expression within the proximal, medial, and distal femurs of HUR vs. AMB mice. Micro-CT also failed to detect any significant difference in endpoint measures of MAT volume (Supplementary Fig. 7h) and ELISA showed no difference in serum leptin levels (Supplementary Fig. 7i) between treatment groups. Together, these data demonstrate that an equivalent period of reloading is sufficient to reverse HU-induced alterations to organismal and BM physiology and cortical bone density but does not fully restore trabecular micro-architecture.

**Fig. 6:**
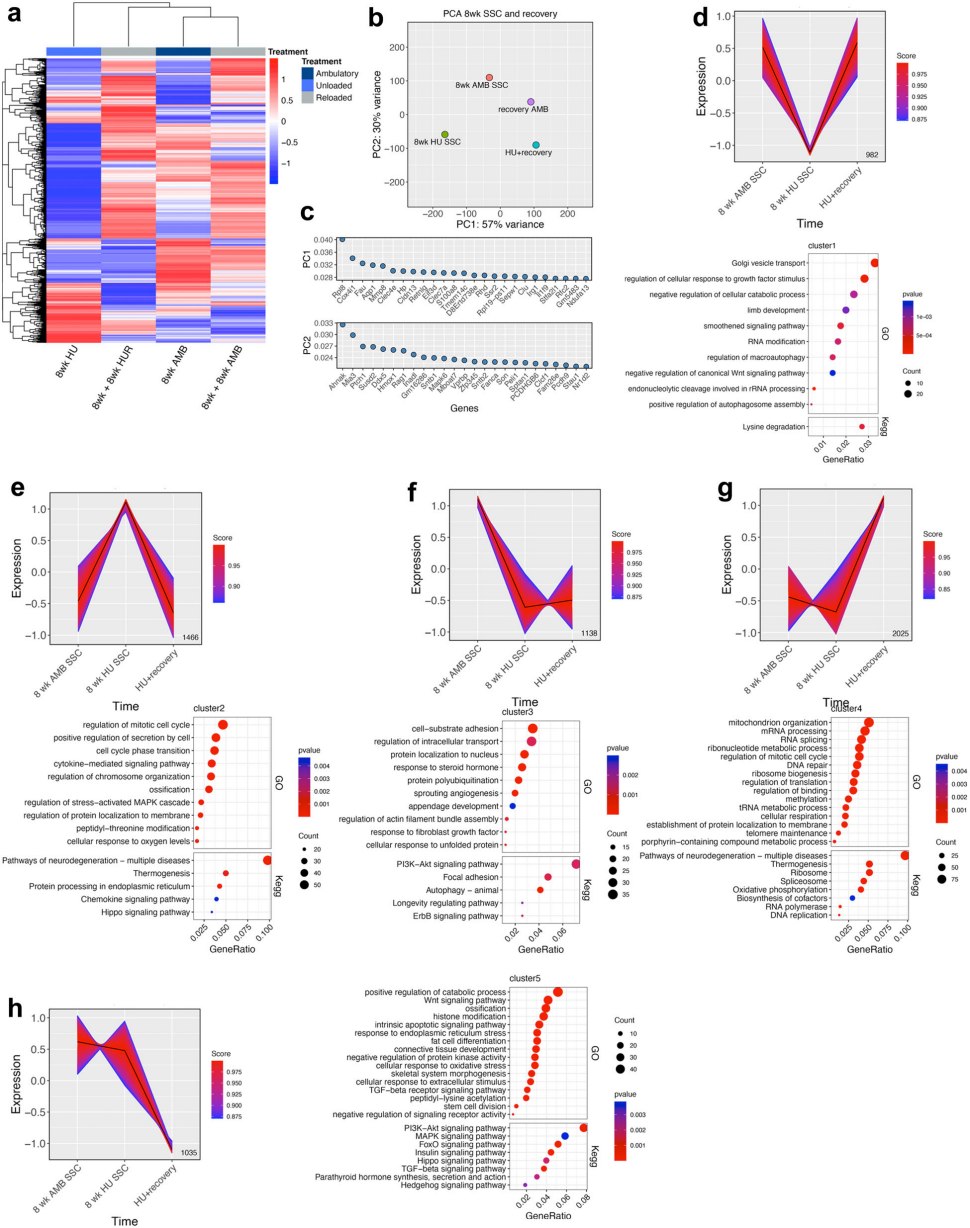
HU and HUR result in temporal and dynamic changes in the SSC transcriptome. **a** Heat map of the top 1000 DEGs from RNA-seq analysis of LEPR^+^ SSCs from AMB, HU, and HUR mice. Colors correspond to per-gene *Z*-score computed across each row. **b**, **c** PCA analysis (**b**) of data from **a** and genes contributing to PC1 and PC2 (**c**). **d**–**h** Gene expression patterns identified by K-means clustering of RNA-seq data from **a** and corresponding GO terms (upper dot plot) and KEGG pathways (lower dot plot) based on *p*-value and gene count identified by gene enrichment from each cluster.

Lastly, we performed bioinformatic analysis of RNA-seq data from all SSC cohorts to resolve dynamic changes in gene expression during HU and HUR. Hierarchical clustering of these data segregated SSCs based on experimental paradigm, with AMB controls more closely related to populations recovered from HUR than 8wk HU mice (Fig. 6a). PCA analysis also revealed large variations among all SSC samples (Fig. 6b), indicating that RNA-seq resolves both age-dependent and unloading/reloading specific impacts on the SSC transcriptome. These data also indicate that HUR does not restore the HU SSC transcriptome to an AMB state. Differences between HU and HUR SSCs were largely attributed to PC1 and its corresponding genes mapped to the GO terms “ammonium transmembrane transporter activity,” “response to external stimuli,” and “extracellular space,” whereas PC2 genes mapped to “calmodulin binding,” “carbohydrate binding,” “negative regulation of cell division,” and “glycoprotein complex” (Fig. 6c).

K-means clustering of these data identified five independent gene expression profiles that depict pseudo-time-resolved changes in the transcriptional program of SSCs from AMB, HU, and HUR mice. EnrichedGO analysis of genes comprising cluster 1, which were downregulated during HU, mapped to terms related to “golgi vesicle transport,” “cellular response to growth factor stimulus,” “limb development,” “negative regulation of WNT signaling pathway,” and several terms related to autophagy and RNA processing (Fig. 6d). KEGG analysis of this gene set returned the term “lysine degradation,” which is often associated with stress response pathways in cells. Genes in cluster 2, which were upregulated by HU, included numerous enrichGO terms related to cell cycle regulation and mitosis, cell secretion, cytokine signaling, ossification, and stress-activated MAPK cascade, and KEGG pathways related to neurodegeneration, thermogenesis, chemokine signaling, and protein processing in the endoplasmic reticulum (Fig. 6e). Together, these gene profiles indicate that HU is perceived as a stressor in SSCs and perturbs niche functions by altering cytokine signaling/secretion and pathways regulating ossification. GO terms related to cell cycle phase transition and mitotis are also consistent with a quiescent phenotype as cells must exit the cell cycle to enter quiescence. Genes in cluster 3, which represent those downregulated in both HU and HUR, mapped to enrichGO terms related to cell-substrate adhesion and actin assembly, intracellular transport, response to steroid hormone and unfolded protein, sprouting angiogenesis, and appendage development, whereas KEGG pathways including focal adhesion, phosphatidyl inositol 3-kinase (PI3K)-AKT and ErbB signaling, and longevity regulated pathway (Fig. 6f). These findings suggest that HU induces localized changes in niche architecture that are not reversed by HUR, and negatively impact both bone anabolic and angiogenic pathways important for bone growth. Alternatively, enrichGO terms related to cluster 4, which included genes upregulated in response to HUR, included terms related to increased cellular activity, such as DNA/RNA modification, cellular metabolic processes, mitochondrial organization, and cellular respiration, whereas KEGG pathways reflected many of the same biological processes (Fig. 6g). Lastly, genes in cluster 5 that were downregulated in HUR mapped to enrichGO terms related to stem cell division, ossification, connective tissue development, skeletal system morphogenesis, and histone modification, and KEGG pathways implicated in skeletogenesis, such as MAPK, PI3K-AKT, FoxO, Hedgehog, transforming growth factor-β, and parathryoid hormone signaling (Fig. 6h). Therefore, although HUR appears to induce exist of SSCs from quiescence and enhance their metabolic activity, it fails to restore pathways implicated in skeletogenesis and ossification.

## Discussion

A large body of literature exists describing impacts of simluated microgravity on rodent skeletal biology. However, interpretation of this literature is complicated by the fact that studies vary in the duration of disuse, often lack a recovery phase, quantify impacts on skeletal pathology or marrow adiposity but not both, and fail to interrogate systemic vs. BM specific impacts^61,62^. Herein, we employed a multi-modal approach to interrogate impacts of HU and recovery on animal physiology, skeletal pathology, and BM signaling, and correlated them with time-resolved changes in the transcript profile of prospectively isolated SSCs and culture-expanded MSCs. A unique aspect of this study was that mice were exposed to 8 or 14 weeks of continuous HU to more realistically model long-term immobility in disabled/injured individuals and microgravity exposure in astronauts. This approach revealed that muscle atrophy observed after 8wk remained stable by 14wk of HU, which is consistent with other studies showing brief intervals of HU induces rapid onset of muscle wasting^63–65^. In contrast, bone loss was progressive in response to prolonged HU, which deviates from an earlier study showing that muscle atrophy and bone loss in HU are interrelated^66^. We did not quantify levels of irisin, a muscle myokine with bone anabolic activity^67–69^, which is downregulated in HU^70^, or the atrogene IL-6^71,72^, which is elevated in HU and also implicated in bone loss^73,74^. Therefore, we cannot rule out effects of these muscle derived factors on bone loss during prolonged HU. Although the underlying mechanisms responsible for muscle atrophy are complex, it is largely reversed following an equivalent period of reloading^75–77^, which was evident in our study.

Prolonged HU also induced significant lipodystrophy in mice as evidenced by significantly decreased body fat mass, decreased serum leptin levels, increased lean body mass, and increased MAT volume when compared to AMB controls. Accordingly, serum leptin levels did not correlate with MAT accumulation as reported previously in high-fat-diet-fed mice^26^. To account for this paradoxical result, we evaluated leptin protein levels in BM via IHC, which was significantly upregulated in response to HU, and both BM leptin and MAT volume were diminished in response to reloading while serum leptin levels were increased. Therefore, MAT volume changes in response to HU correlated with BM and not systemic leptin levels, which suggest that BM adipocytes, which are known to secrete leptin^78^, serve as a local source of this hormone. While the effects of leptin on bone are complex^79–81^, leptin overproduction is known to inhibit bone formation^82,83^. There also exists a strong association between osteoporosis and increased MAT volume due to the anti-osteogenic activity of fatty acids, adipokines and receptor activator of nuclear factor-κΒ ligand secreted by adipocytes^84–87^. Therefore, altered local leptin expression may drive MAT accumulation in response to HU. IHC analysis also revealed that HU also upregulated BM expression of TH, a marker of catecholamine neurons, which is consistent with previous reports linking increased sympathetic tone to DO^41,45^. Consistent with these findings, HU-induced bone loss has been shown to be ameliorated by the concomitant treatment with β-blockers^19,41,88^ and mice lacking expression of β_2_-adrenergic receptor exhibit a high bone mass phenotype^45^. Increased sympathetic tone has also been linked to lipolysis^89^ and as such observed increases in BM TH and MAT volume in HU mice are paradoxical. However, BM adipocytes were recently shown to be insensitive to the lipolytic activity of isoprenaline, a β-adrenergic agonist, due to their low expression levels of monoacylglycerol lipase^90^, which may explain increases in both TH protein and MAT.

Recent studies have begun to address the impacts of HU and HUR on the transcriptome of SSC in BM. For example, Li et al.^91^ discovered that administration of an EIF2α dephosphorylation inhibitor to mice undergoing 14d of HU led to improvements in bone loss and increased the number of BM CFU-Fs, which provides a readout of SSC frequency. In addition, Hu et al.^92^ found that Thy1^+^ITGA5^+^ITGB1^+^CD90.1^−^CD29^−^CD49e^−^CD11b^−^CD45^−^ cells from mouse BM, which share some markers with SSCs, decreased during HU, and that this effect could be partially ameliorated by a custom device that stimulated fluid flow within the unloaded limbs. However, this effect was short lived and impacts on bone physiology were not assessed^92^. Another recent study found that 2wk of HU in 2-month-old mice led to a decrease in Lin^-^LEPR^+^Oln^+^ BM SSCs, which were shown to be perivascular and overlap with cell types that exhibit mechanosensitive properties^93^. Similarly, we also observed a downward trend in the frequency of Lin^-^LEPR^+^ SSCs in BM following HU, and showed these cells exhibit dynamic and time-resolved changes in gene expression in response to prolonged HU and following recovery, which paralleled alterations in skeletal integrity and BM niche signaling. Specifically, our data indicate that transcriptional responses to 8 and 14wk of HU are indicative of a deepening state of cellular quiescence based on downregulation of metabolic and genetic information processing genes/pathways, which are consistent with previous studies showing that simulated microgravity suppresses proliferation of muscle satellite cells^94^, osteoblasts^95^, and cultured HSCs^96^, and is in agreement with studies describing different functional phases of quiescence in muscle stem cells based on their metabolic status and responsiveness to tissue injury^97^. Our data further indicate that SSCs undergo stem cell maintenance in response to HU, which mirrors observed adaptations to mechanical changes observed in other stem cells^52,98^. Furthermore, although leptin overproduction is known to inhibit bone formation^82,83^ and skew SSC bifurcation toward adipogenesis^26^, we did not observe HU-induced changes in SSC adipogenic pathways, leaving the biogenesis of MAT in HU indeterminate. Therefore, it is interesting to speculate that SSC bifurcation to fat may occur at early stages of HU or that MAT accumulation is driven by maturation of existing adipogenic progenitors. Our pseudo-time course analysis further revealed that SSCs exit quiescence in response to reloading but fail to re-express critical bone anabolic pathways indicative of impaired osteogenic potential. The normalization by HUR of systemic and local factors but not trabecular micro-architecture indicates that physical disruption of the BM niche during HU leads to enduring cell-intrinsic defects in SSCs. Together, these results add to evidence from aging, obesity and diabetes models showing that BM adiposity and peripheral neuropathy impact SSC function^26,99–101^.

Unlike SSCs, we demonstrate that MSCs are largely insensitive to HU, which differs from earlier reports that simulated and non-simulated microgravity negatively impacts their growth, differentiation^31,36,102^, and transcription^62^. An important difference between these and our studies is that we enriched MSCs to high purity via immuno-depletion, whereas others evaluated heterogeneous BM cells with low MSC abundance. Alternatively, in vitro studies employing homogeneous MSCs demonstrated simulated microgravity globally represses gene expression, cell cycle, and calcium signaling^103^, and spaceflight represses osteogenesis‐related pathways and induces adipogenic regulators^104^. Therefore, although transcription in cultured MSCs is directly altered by microgravity, impacts of HU in live animals on BM MSC transcription are largely erased following purification and culture adaptation.

Gene expression studies undertaken in this work focused solely on marrow resident Lin-LEPR+ SSCs initially identified by Zhou et al.^24^. Although several cell populations have been identified in marrow either by lineage tracing or cell sorting to retain properties of SSCs, we focused on the Lin-LEPR+ fraction, as it was shown to function as a direct precursor of osteoblasts and adipocytes in adult BM by lineage tracing, and lineage bifurcation of these cells was also shown to be responsive to local and systemic factors, such as high-fat diet feeding and bone fracture. Therefore, we hypothesized this population would also be sensitive to HU and HUR, which we demonstrated empirically. However, it remains possible that other SSC populations exist in marrow that are equally, more, or less responsive to HU and HUR, and our data provides a framework to make direct comparisons to these populations. As SSCs lie in close proximity to trabeculae in BM^24,105^, we propose that HU-induced alterations in BM niche signaling and erosion of trabecular micro-architecture repress bone anabolic pathways in SSCs resulting in reduced osteogenic activity. Although additional mechanistic studies are needed to related gene expression changes to impacts on SSC differentiation, the self-maintenance gene signature identified herein may represent pro-survival factors and/or therapeutic targets for safeguarding SSCs against detrimental impacts of DO. Therefore, we also propose that therapeutics that preserve the BM niche and SSC osteogenic and metabolic function during disuse may prevent bone loss and speed recovery following return to normal weight-bearing activity.

## Methods

### Mice

All mice (C57Bl6 males; Jackson Labs) were 3 months old at the start of experimentation. For HU, mice were anesthetized with isoflurane (2% to effect), fitted with a 23 G sterilized surgical steel tail ring through the fifth or sixth intervertebral disc space and single-housed at 21 °C. Baseline body weight and composition were used to randomize mice into the two study groups (AMB or HU) such that the mean body weight and body fat % between groups were not statistically different. After 5–7 days, mice were continuously suspended via the tail ring at ∼30° head‐down angle so their hind limbs remained elevated above the cage floor for 8 or 14 weeks. AMB controls were subject to mock tail ring surgery and single-housed. Body weight was monitored weekly and blood was drawn at regular intervals for serum profiling from the submandibular vein. A metal hook stand was used to prevent hindlimb loading during body weight measurements. To prevent hindlimb loading, mice were given daily rations of standard rodent diet pellets in excess of their caloric needs secured to the bottom of the cage floor away from the range of motion of the hindlimbs. To maintain hydration mice were given daily rations of hydrogel (ClearH2O 70-01-1082) mixed with water-soaked standard rodent diet pellets in dishes similarly secured to the bottom of the cage. Hydration was further supplemented with subcutaneous saline injections after blood collections and as needed. AMB mice were maintained under the same dietary and hydration protocol. Some mice subjected to 8 weeks of HU were relieved from tail suspension and housed for an additional 8 weeks of recovery (HUR). During HUR, mice were given standard rodent diet ad lib and cage water. At the study endpoint, mice were killed, blood was drawn by cardiac puncture, and other tissues collected. Power analysis of initial data indicated that four mice per group was sufficient to achieve statistical significance of micro-CT bone measurements. Therefore, group sizes were restricted to a minimum of four mice and each experiment consisted of two independent trials. Data from the two trials were grouped where indicated. No exclusion criteria were established or needed. All procedures were approved by the Institutional Animal Use and Care Committee of Scripps Florida.

### Stem cell isolation and characterization

MSCs were isolated from BM using a culture-based immuno-depletion protocol as previously described with modification^51,106^. Briefly, BM was extruded from the femurs and tibiae of HU or AMB mice, enzymatically digested for 30 m at 37 °C using a cocktail of 500 μg/mL Liberase DL (Roche 05401160001) and 400 U/mL DNAseI (Sigma D4527), and plated in 15 cm Nunclon Delta tissue culture dishes (Thermo Fisher 150468). Media comprised MEM-alpha GlutaMax (Gibco 32561-037), 10% fetal bovine serum (FBS) (Sigma 12303 C) and 100 U/mL of Penicillin/Streptomycin (Gibco 15140-122). After 7 days in culture, plastic-adherent cells were collected by trypsinization with 0.25% Trypsin EDTA (Gibco 25200-056), washed using serum-free media, and immune-depleted by magnetic streptavidin-labeled Dynabead (Invitrogen 11206D) separation using biotinylated anti-CD11b (BD 553309), anti-CD34 (BioLegend 119304), and anti-CD45 (BD 553078) antibodies. Immediately after immuno-depletion, MSCs from three mice per group were pooled, pelleted, and RNA extraction performed using the Quick-RNA MiniPrep Kit (Zymo Research R1054).

To enrich SSCs, marrow was extruded from femurs and tibia of individual mice, digested for 30 m at 37 °C with Liberase DL (Sigma-Aldrich 05401160001) and DNAseI (Sigma D4527), red blood cell lysis was performed using ACK lysis buffer, and cells resuspended in Hank’s buffered salt solution (without calcium and magnesium). Marrow cells were stained with CD11b (BD 553309, 1 : 200), anti-CD34 (BioLegend 119304, 1 : 200), and anti-CD45 (BD 553078, 1 : 200), and then labeled cells were removed using 10 μL M280 Streptavidin Magnetic beads (Invitrogen 11206D). The remaining cells were washed and labeled with CD16/32 Fc block (BD 553142, 1 : 40), FITC anti-Ter119 antibody (Tonbo 35-5921, 1 : 100), FITC anti-CD31 antibody (BD 553372, 1 : 50), FITC anti-CD45 antibody (BD 553078, 1 : 100), biotinylated anti-LEPR antibody (R&D BAF497, 1 : 33), donkey-anti-goat Alexa 647 antibody (Invitrogen A21447, 1 : 40), and propidium iodide (BD 51-66211E, 1 : 100). The Lin^−^LEPR^+^ SSC fraction was then isolated by FACS using a BD FACS Aria at a flow rate of <10,000 cells/s using a 100 μM nozzle. Cells from three or four mice per group were pooled and total RNA isolated using the Quick-RNA MiniPrep Kit (Zymo Research R1054). To assess SSC purity, CFU-F activity was quantified in the Lin^+^LEPR^+^ and Lin^−^LEPR^+^ fractions by plating equivalent numbers of isolated cells immediately after sorting and culturing for 7–10 days at 37 °C in 5% O_2_ and 5% CO_2_. In addition, standard colony assays were performed using cell fractions from each step of the isolation procedure (whole BM, post RBC lysis, Lin^+^LEPR^+^, Lin^+^LEPR^−^, and Lin^−^LEPR^+^). Briefly, 10,000 cells in 100 μL RPMI 1640 (Lonza 12-115F) with 2% FBS were added to 1.2 mL of thawed mouse methylcellulose with growth factors (R&D Systems HSC007) and thoroughly mixed by vortexing. The cell/methylcellulose mixture (1.1 mL) was plated into a 35 mm Nunclon Delta tissue culture dish (Thermo Fisher 150460) using a syringe outfitted with a 16 G needle and were maintained at 37 °C in 5% O_2_ and 5% CO_2_. After 10 days, BM colonies were hand scored.

### Metabolic profiling

At the indicated time points, blood was drawn from the submandibular vein (<100 µL) or via cardiac puncture (after killing only) and collected in uncoated 1.5 mL tubes. After clot formation, the tubes were centrifuged 5 min at 1000 × *g*, serum was decanted, and stored at −80 °C. BUN (Roche 11729691), triglycerides (Roche 20767107), LDL (Roche 03038866), glucose (Roche 04404483), cholesterol (Roche 03039773), ALP (Roche 03333752), AST (Roche 20764949), ALT (Roche 20764957), and HDL (Roche 04399803) quantification assays were determined by the Metabolic Core Facility at Scripps Florida using the Cobas c311 Clinical Chemistry Analyzer (Roche Diagnostics). The Minispec LF-50/mq 7.5 NMR (Bruker Optics) was used to determine lean body mass, fluid mass, and fat mass of whole mice. Leptin levels were quantified using the Quantikine Mouse/Rat Leptin ELISA Kit (R&D Systems MOB00) and corticosterone levels were determined by LC/MS in the DMPK Core Facility at Scripps Florida. Briefly, 10 µL of each serum sample or carbamazepine standard was injected into the UFLC XR (Shimadzu) with attached BetaSil C18 5 μm 50 × 2.1 mm analytical column (Thermo Fisher). The mobile phase was water with 0.1% formic acid and acetonitrile in 0.1% formic acid, and was delivered at a rate of 0.35 mL/min. The triple quadrupole API 5500 Qtrap (SCIEX) mass spectrometer was used in electrospray ionization, positive polarity mode. Multiple reaction monitoring was used to detect analytes with *m*/*z* of 347.2/91 corresponding to corticosterone and 237.2/194.1 corresponding to carbamazepine.

### Micro-CT analysis

The tibia of one hindlimb per mouse was carefully removed at the knee, keeping the proximal tibia and muscles intact. This tibia was stored in 10% neutral buffered formalin and shipped to the University of Toledo. After shipping, tibiae were dissected, fixed overnight in 4% paraformaldehyde, and stored in 70% ethanol at 4 °C. Bone and MAT scans were conducted using a µCT35 micro-CT system (SCANCO Medical AG) with the X-ray source operating at 70 kVp and 114 μA energy settings, and recording 500 projections/180° acquired at 300 ms integration time using. Scans of trabecular bone in the proximal tibia consisted of 300 slices covering a distance of 2.1 mm from the growth plate, and scans of cortical bone consisted of 57 slices covering 0.4 mm of the tibia midshaft. Segmentation of trabecular bone images was conducted on 200 total slices beginning 10 slices from the growth plate following manual contouring (optimized gray-scale threshold of 220 per mille, equivalent to 3313 Hounsfield units, or μ of 1.76). Segmentation of cortical bone images are conducted on the entire image stack of 57 slices following semi-automatic contouring (260 per mille threshold, 3673 Hounsfield Units). Trabecular and cortical bone morphometric parameters were calculated directly from voxel values with a 7 μm nominal resolution voxel for trabecular bone and 12 μm for cortical bone. Lipid distribution and volume was conducted on decalcified bone specimens stained for 2 h in 0.1 M sodium cacodylate buffer (pH 7.4) with 2% osmium tetroxide. Images of lipid deposits were acquired at 12 µm resolution and quantified directly from voxel volumes from whole tibiae. The data shown from bone and MAT scans were generated using Evaluation Program V6.5-3 (Scanco Medical AG) and analyzed using recommended guidelines^107^.

### Mouse behavior studies

Behavior studies were performed on mice at 6.5 weeks of HU in Noldus PhenoTypers (Noldus Information Technology) at 21 °C using the same rationed food/hydrogel regimen described above. The 30 × 30 × 35 h cm clear polymethylmethacrylate cage lids are outfitted with infrared lighting and an IR-sensitive camera that allows for non-invasive recording of behavior. Seven cages of single-housed mice were divided among four shelves and visually separated from each other. Room lights were programmed to maintain a 12 : 12 light : dark cycle with lights on at 07:00 AM. Mice were introduced into the cages just before the dark cycle and all health checks and feedings were performed at this time on subsequent days. Care was taken to provide white noise and minimize foot traffic in the observation room. Four cages were retrofitted to accommodate the tail ring rig for the HU group. The rig did not interfere with the IR camera field of view. The remaining three cages were left unaltered to give AMB mice free mobility. IR videos were recorded starting with the dark cycle each day for 20 h. After 2 days of habituation to the PhenoTyper cages, video was collected each day through day 10. Mouse behavior in the recordings was analyzed using EthoVision XT (Noldus Information Technology). Distance moved and activity state were compared between AMB and HU mice. Activity vs. inactivity states were determined by quantifying the changes in pixel intensity between frames with user-defined thresholds established from video samples from the days collected.

### Histological staining

Femurs (one each from two mice per group for 8wk HU and both femurs for one mouse per group for HUR) were carefully excised and fixed in 10% neutral buffered formalin, decalcified with Immunocal Decalcifier (Statlab 1414-1), processed (Sakura Tissue-Tek VIP5), embedded in paraffin, sectioned at 4 µm, and mounted on plus slides. The slides were stained with Tyrosine Hydroxylase antibody (EMD Millipore, AB152 1 : 500) or Leptin/OB (Novus Biologicals MAB498, 1 : 4000) and the BOND Polymer Refine DAB Detection kit (Leica DS9800) on a Leica BOND-MAX immunostaining platform, dehydrated in graded alcohols, cleared in xylene, and cover slipped with Cytoseal 60 (Thermo Scientific 8310). For each stain, four sections per femur were analyzed. The BM of each IHC section was randomly photographed at 100 × 3 times in each of three regions: proximal (in or near the femoral head), medial (within the shaft of the bone), and distal (in or near the epicondyles). Micrographs were scored using the IHC Profiler^108^ plugin for ImageJ, which allows for non-biased, automated scoring of histological slides. In this method, each DAB-stained pixel is categorized into one of four pre-set pixel intensity bins (High Positive, Positive, Low Positive, Negative) and yields the percent contribution of each bin to the image.

### RNA sequencing

Total RNA was pooled from three or four mice per group to increase yields for library prep. RNA was run on an Agilent 2100 Bioanalyzer RNA pico chip (Agilent Technologies 5067-1513) for quality assessment and quantification, and subsequently concentrated with a SpeedVac Vacuum Concentrator (Thermo Scientific). Libraries were prepared in an RNase-free working environment. For all samples except 14wk AMB and HU MSC (processed as total RNA), poly-adenylated RNAs were selectively isolated from total RNA (~10 ng) using poly-T oligos attached to magnetic beads according to the manufacturer’s guidelines in the NEBNext poly(A) mRNA magnetic isolation module (NEB E7490). Library preparation from enriched mRNA and total RNA was completed with the NEBNext Ultra II Directional RNA kit (NEB E7760). The double-stranded (ds) cDNA was purified with 2.0X Agencourt AMPure XP bead (Beckman Coulter A63881) ratio to retain insert fragments >80 nt. The ds cDNA was end repaired and adenylated at the 3′-end. A corresponding “T” nucleotide on the adaptors was utilized for ligating the adaptor sequences to the ds cDNA. The adaptor ligated DNA was purified using 1.2× bead ratio and PCR amplified using 15 cycles to incorporate a unique barcode and to generate the final libraries. The final libraries were size selected and purified using 1.0× Ampure XP beads to remove any primer dimers and validated on a Bioanalyzer High Sensitivity DNA chip and normalized to 1 nM. After equimolar pooling, the libraries were loaded at a 1.8 pM concentration, and sequenced on a NextSeq 500 (Illumina) with 2 × 40 bp paired-end chemistry. On average, 20–25 million pass filter (base quality score > Q30, suggesting <1 error in 1000 bp) reads were generated.

### RNA-Seq data analysis

All raw RNA-Seq data processing was conducted on the Galaxy^109^ instance at the Scripps Research Institute - Florida. Collected reads were downloaded as raw FASTQ files and quality checks were performed using the FASTQC application^110^. CutAdapt^111^ was used to remove adapter sequences and short reads. Reads were mapped against the NCBI Build 37 mm9 mouse reference genome using the HISAT2^112^ and featureCounts^113^ application packages. Raw read counts were then exported for downstream analysis in the R software package environment. Differential gene expression analysis was carried out in R using the edgeR package^114^. Briefly, low expressing raw reads across samples were filtered out such that the sum of counts was >2 across any given transcript. Raw read counts were normalized with the edgeR calcNormFactors function to generate a model of normalized reads (cpm), which were then filtered to only include protein coding transcripts in subsequent analyses. A dispersion value of 0.2 was used for differential expression exact test analysis. DEGs were used to create ranked ordered lists for biological process GO, GSEA, and KEGG pathway analysis using the clusterProfiler R package^115^. Following term simplification, the top 25 GO terms were displayed. PCA analysis was carried out using the DESeq2 software package^116^. Heatmaps were generated using the Pheatmaps R package^117^. Heatmaps are displayed as a calculated *Z*-score across each row of transcripts and display the top 1000 most highly variable transcripts. The Enhanced Volcano package^118^ was used to generate volcano plots of DEGs. K-means gene expression clustering was performed in R with the cluster package^119^. The number of significant clusters was determined to be five using the sum of squares method. The enrich function in clusterProfiler was used to map genes from each cluster into GO and KEGG pathways. Ontologies with *p* < 0.005 were ranked by degree of term redundancy within the data set and the top 10–20 selected.

### Quantification and statistical analysis

The statistical significance between two independent experimental groups was assessed using a two-tailed, unpaired Student’s *t*-test. The statistical significance among more than two groups was assessed using a one-way ANOVA with multiple comparisons assessed using the Tukey’s test. Significance level was set at *p* ≤ 0.05, unless otherwise indicated. All data represent mean ± SD.

### Reporting summary

Further information on research design is available in the Nature Research Reporting Summary linked to this article.

## Supplementary information

Supplementary Information

Reporting Summary

## Data Availability

The data that support the findings in this study are available from the corresponding author upon reasonable request.

## Code availability

Raw RNA-sequencing data found in this study are available at GEO (Gene Expression Omnibus) database by the accession number GSE156174.
