# Author Correction: Transcriptional responses of skeletal stem/progenitor cells to hindlimb unloading and recovery correlate with localized but not systemic multi-systems impacts

**DOI:** 10.1038/s41526-021-00184-2

**Published:** 2021-12-15

**Authors:** Cori N. Booker, Christopher L. Haga, Siddaraju V. Boregowda, Jacqueline Strivelli, Donald G. Phinney

**Affiliations:** Department of Molecular Medicine, The Scripps Research Institute – Scripps Florida, Jupiter, Florida 33458 USA

**Keywords:** Stem cells, Systems biology

Correction to: *npj Microgravity* 10.1038/s41526-021-00178-0, published online 26 November 2021.

In this article Cori N. Booker and Christopher L. Haga should have been denoted as equally contributing authors. The original article has been corrected.

