# Supplementary material for: Transcriptional responses of skeletal stem/progenitor cells to hindlimb unloading and recovery correlate with localized but not systemic multi-systems impacts
Source: NPJ Microgravity. 2021 Nov 26;7:49. doi: 10.1038/s41526-021-00178-0 (PMC8626488; doi:10.1038/s41526-021-00178-0)

Supplementary Figures and Figure Legends

Supplemental Figure 1. Profiling of toxicity and systemic metabolic parameters in 8wk HU and AMB mice. a-g Time course of alanine aminotransferase (ALT, a), aspartate aminotransferase (AST, b) blood urea nitrogen (BUN, c) total blood glucose (d), total cholesterol (e), triacylglycerol (TAG, f), and high-density lipoprotein (HDL, g) levels in peripheral blood of non-fasted 8wk HU mice and AMB controls wherein numbers in parenthesis are days post-HU, which began at day 1 (n = 4-6 mice/group). (n = 4-6 mice/group). Data are mean ± SD. P-values are by Student's t test of AMB vs HU mice at each time point.

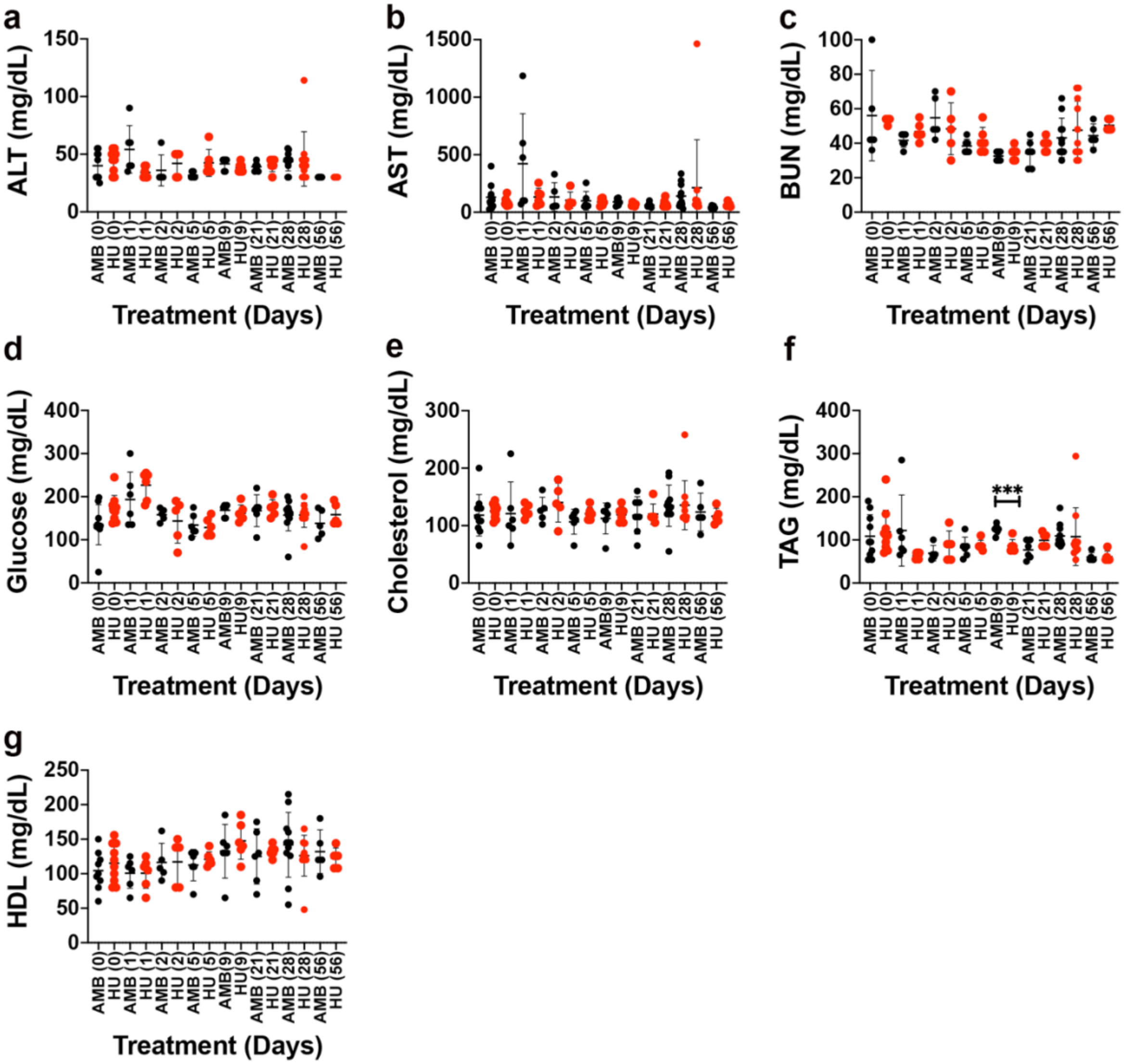

Supplementary Figure 2. Mice subjected to 8wk of HU exhibited elevated corticosterone levels and increased activity. a Time course of serum corticosterone levels in 8wk HU and AMB mice wherein numbers in parenthesis are days post-HU, which began at day 1 (n = 6-14 mice/group). **b-d** Behavior assessments showing total distance traveled (b), distanced moved per unit area of accessible space (c) and total time mice spent moving (d) by mice over a 10d observation period (n = 4mice/group). **e** Movement of individual mice over a 20 h period on day 8 of observation. Data are mean ± SD. P-values in a, b are by Student's t test and in c-e by one-way ANOVA and Tukey post-hoc test.

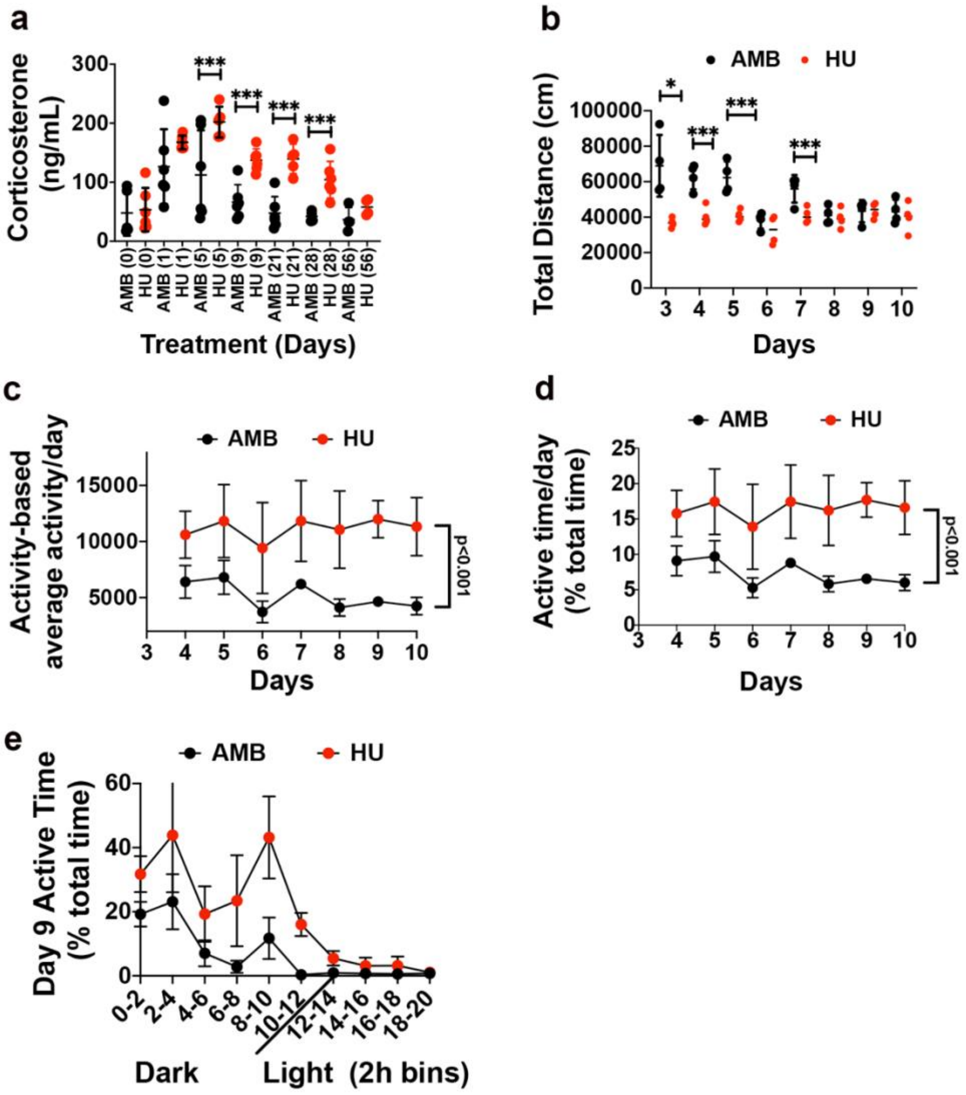

Supplementary Figure 3. Isolation of Lin$^-$LEPR$^+$ SSCs from BM. a-d Representative dot plots from flow cytometric analysis of whole bone marrow cells showing forward vs. side scatter (a), forward scatter height and width (b), viability staining with propidium iodide (c), and gating strategy used to identify SSCs after staining with antibodies against lineage-specific markers (CD31, CD45, Ter119) and LEPR (d). **e** CFU-F activity in Lin$^+$LEPR$^+$ and Lin$^-$LEPR$^+$ populations from (d). Data are mean ± SD from experiments repeated four times and p-value by Student's *t* test. Inserts are photomicrographs of Lin$^+$LEPR$^+$ and Lin$^-$LEPR$^+$ cells stained with Geimsa. **f** Colony forming activity of bone marrow prior to (BM) and after RBC lysis (RBC lysis) and of Lin$^+$LEPR$^-$, Lin$^+$LEPR$^+$ and Lin$^-$LEPR$^+$ populations recovered from (d). Data are mean ± SD from experiments performed in triplicate. **g** Total SCCs yields from pooled BM (n = 3 mice/preparation). Data are mean ± SD (n = 4 mice). and p-value by Student's t test.

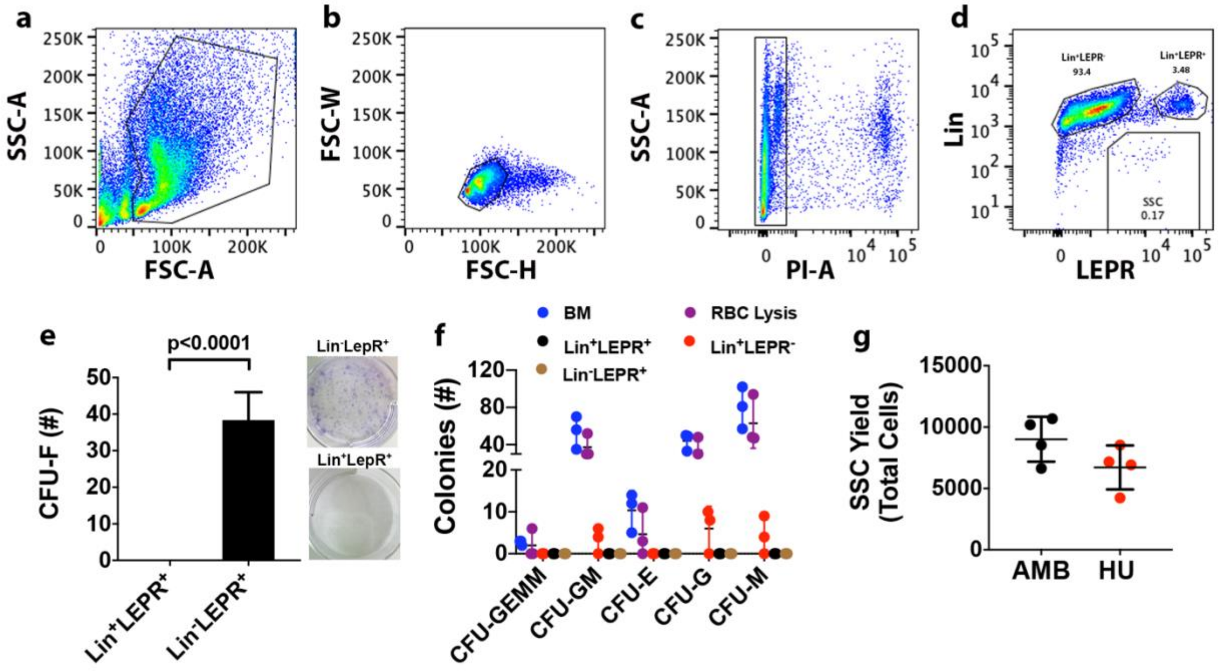

Supplemental Figure 4. SSCs downregulate metabolic pathways while MSC upregulate growth regulator pathways in response to 8wk of HU. a Gene sets contributing to PC1 and PC2 based on PCA analysis of RNA-seq data obtained from SSCs and MSCs harvested from 8wk HU mice and their respective AMB controls. **b** Top GO terms based on *p*-value (colored scale) identified by GSEA of DEGs in MSCs from 8wk HU vs. AMB mice. Red arrows indicate upregulated gene sets corresponding to each GO term and gene counts are represented by circle size. **c** KEGG pathway analysis of DEGs from RNA-seq data comparing SSCs from 8wk HU vs. 8wk AMB mice. Colors represent fold change of each designated gene and size represents number of genes within each pathway.

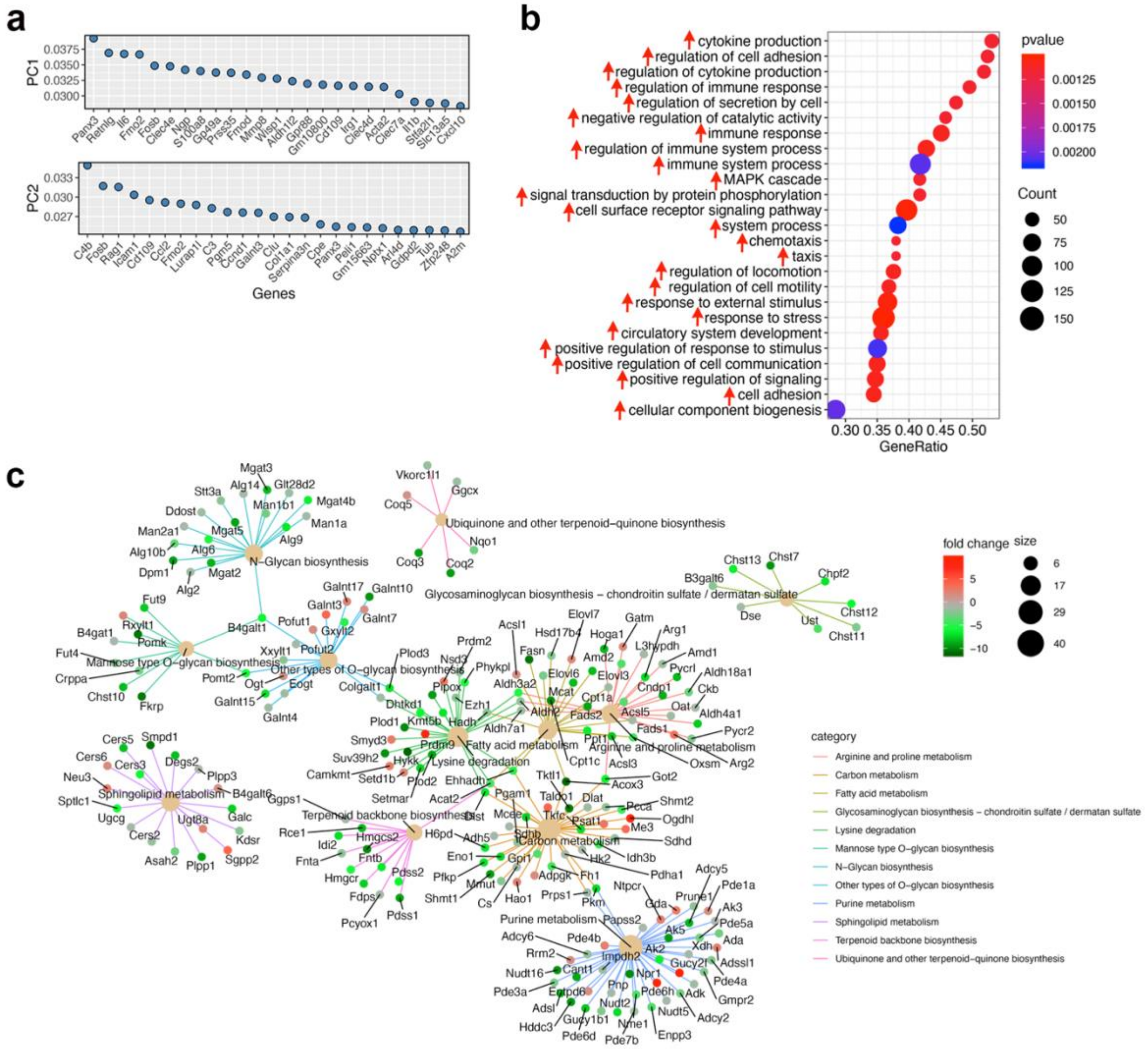

Supplemental Figure 5. SSCs exhibit adaptive responses to prolonged HU. a Heat map of the top 1000 DEGs from RNA-seq analysis of $Lin^{-}LEPR^{+}$ SSCs and MSCs from 14wk HU vs. AMB mice. Colors correspond to per-gene Z-score computed across each row. **b** PCA analysis of data from (a). **c** Genes contributing to PC1 and PC2 from (b). **d, e** Volcano plots showing Log2 fold-change (FC) values for DEGs and their corresponding p-values in SSCs (d) and MSCs (e). Red circles denote DEGs with Log2 FC>2. **f** Violin plot of data from (d and e) showing DEGs up or down regulated by >Log2-fold in SSCs or MSCs in response to HU. **g** Venn diagram showing overlap of up and down regulated DEGs (>Log2-fold) in SSCs and MSCs. **h** Top GO terms based on p-value identified by GSEA of DEGs in SSCs from 14wk HU vs. AMB mice. Red and black arrows indicate up and downregulated gene sets, respectively, corresponding to each GO term. **i, j** KEGG pathway analysis showing fold change of DEGs within gene regulatory networks related to human diseases (i) and genetic information processing (j).

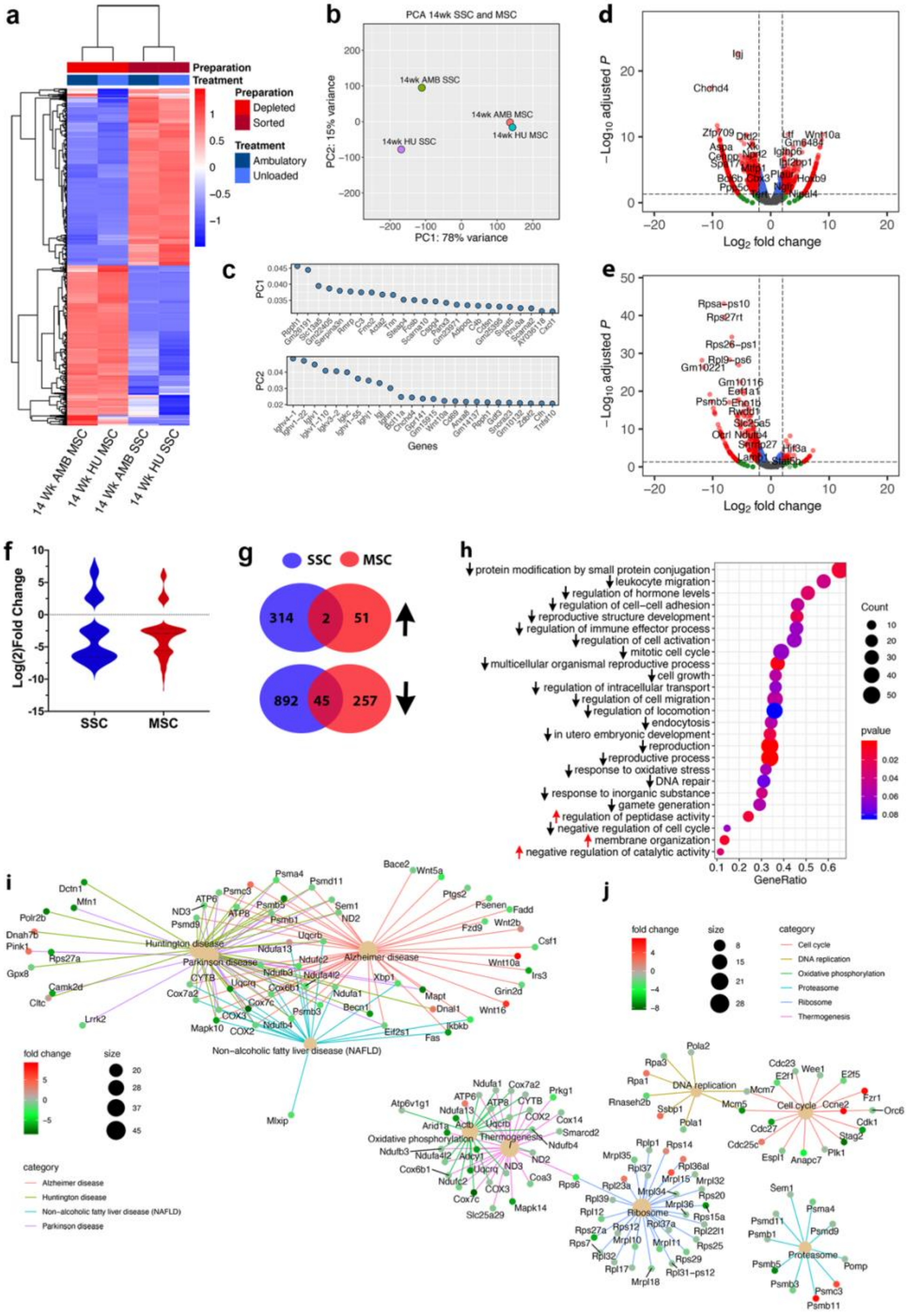

Supplemental Figure 6. Profiling of toxicity and systemic metabolic parameters in 14wk HU mice. a-d Time course of total blood glucose (a), total cholesterol (b), TAG (c), and HDL (d) levels in peripheral blood of HUR and AMB mice wherein numbers in parenthesis represent days post-HUR, which began on day 1 (n = 4-6 mice/group). **e** Corticosterone levels in AMB and HUR mice wherein numbers in parenthesis are days post HU or HUR (n = 4-5 mice/group). Data are mean ± SD and p-values in a-d are by Student's t test (AMB vs HU mice at each time point) and in e by one-way ANOVA and Tukey post-hoc test.

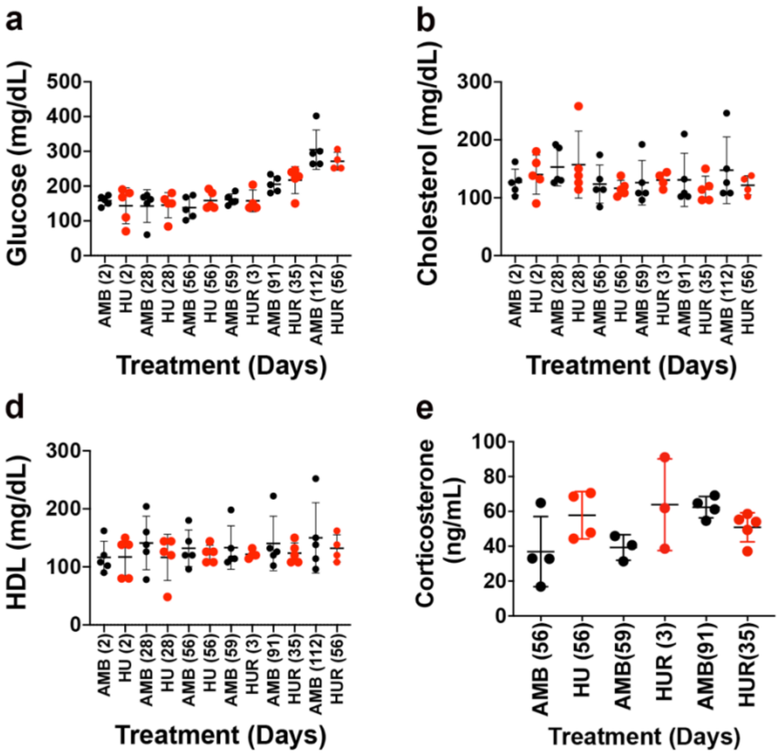

Supplemental Figure 7. HUR normalizes BM leptin and TH protein levels. a Representative photomicrographs of femoral bone tissue sections from AMB and HUR mice stained with HE or antibodies against leptin or TH (100X, scale bar = 50μm). **b-g** Semi-quantitative analysis of leptin (b-d) and TH (e-g) protein levels in the proximal (b, e), medial (c, f) and distal (d, g) femur of AMB and HUR mice (n=2 femurs/group). **h** Micro-CT based volumetric measurements of MAT in AMB and HUR mice (n = 8 mice/group). **i** Endpoint measures of serum leptin levels in HU and HUR mice and their respective AMB controls (n = 8-10 mice/group). Data are mean ± SD. P-values in b-h are by Student's t test (AMB vs HU mice) and in i by one-way ANOVA and Tukey post-hoc test.

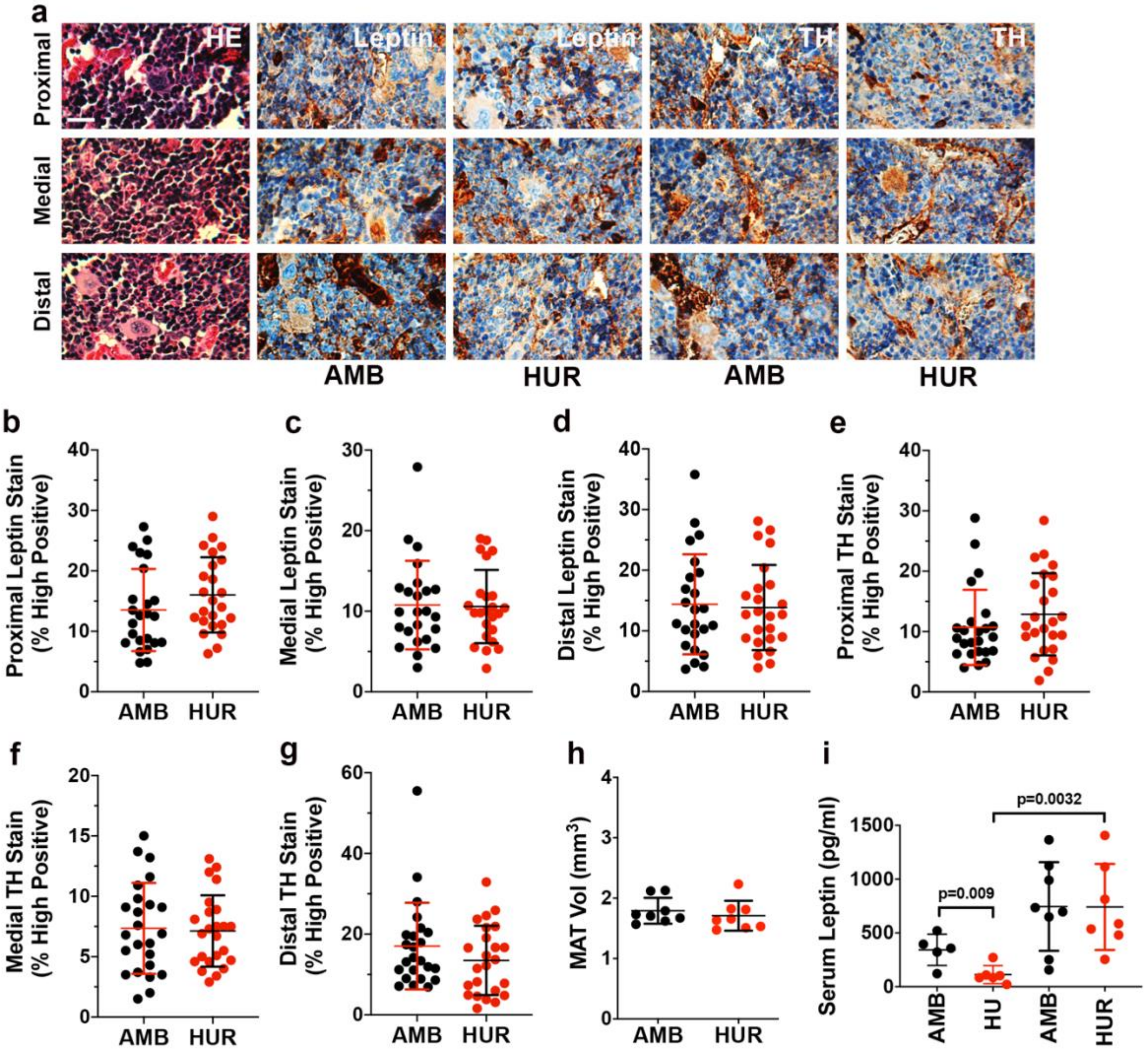

Supplement: Supplementary file 1 — Supplementary Information [file 41526_2021_178_MOESM1_ESM.pdf]
