# Supplementary material for: Transcriptional responses of skeletal stem/progenitor cells to hindlimb unloading and recovery correlate with localized but not systemic multi-systems impacts
Source: NPJ Microgravity. 2021 Nov 26;7:49. doi: 10.1038/s41526-021-00178-0 (PMC8626488; doi:10.1038/s41526-021-00178-0)
Supplement: Supplementary file 2 — Reporting Summary [file 41526_2021_178_MOESM2_ESM.pdf]

nature portfolio

Corresponding author(s): Donald G Phinney, PhD

Last updated by author(s): Oct 25, 2021

Reporting Summary

Nature Portfolio wishes to improve the reproducibility of the work that we publish. This form provides structure for consistency and transparency in reporting. For further information on Nature Portfolio policies, see our Editorial Policies and the Editorial Policy Checklist.

Statistics

For all statistical analyses, confirm that the following items are present in the figure legend, table legend, main text, or Methods section.

n/a	Confirmed	
☐	☒	The exact sample size (*n*) for each experimental group/condition, given as a discrete number and unit of measurement
☐	☒	A statement on whether measurements were taken from distinct samples or whether the same sample was measured repeatedly
☐	☒	The statistical test(s) used AND whether they are one- or two-sided<br>*Only common tests should be described solely by name; describe more complex techniques in the Methods section.*
☐	☒	A description of all covariates tested
☐	☒	A description of any assumptions or corrections, such as tests of normality and adjustment for multiple comparisons
☐	☒	A full description of the statistical parameters including central tendency (e.g. means) or other basic estimates (e.g. regression coefficient) AND variation (e.g. standard deviation) or associated estimates of uncertainty (e.g. confidence intervals)
☒	☐	For null hypothesis testing, the test statistic (e.g. *F*, *t*, *r*) with confidence intervals, effect sizes, degrees of freedom and *P* value noted<br>*Give P values as exact values whenever suitable.*
☒	☐	For Bayesian analysis, information on the choice of priors and Markov chain Monte Carlo settings
☐	☒	For hierarchical and complex designs, identification of the appropriate level for tests and full reporting of outcomes
☒	☐	Estimates of effect sizes (e.g. Cohen's *d*, Pearson's *r*), indicating how they were calculated

Our web collection on statistics for biologists contains articles on many of the points above.

Software and code

Policy information about availability of computer code

Data collection	No software package was used
Data analysis	FASTQC, CutAdapt, HISAT2, featureCounts package, edgeR, clusterProfiler, DESeq2, Enhanced Volcano package, Image J IHC Profiler.

For manuscripts utilizing custom algorithms or software that are central to the research but not yet described in published literature, software must be made available to editors and reviewers. We strongly encourage code deposition in a community repository (e.g. GitHub). See the Nature Portfolio guidelines for submitting code & software for further information.

Data

Policy information about availability of data

All manuscripts must include a data availability statement. This statement should provide the following information, where applicable:

- Accession codes, unique identifiers, or web links for publicly available datasets
- A description of any restrictions on data availability
- For clinical datasets or third party data, please ensure that the statement adheres to our policy

Raw RNA sequencing data are available at GEO database, accession number GSE156174.

Field-specific reporting

Please select the one below that is the best fit for your research. If you are not sure, read the appropriate sections before making your selection.

☒ Life sciences ☐ Behavioural & social sciences ☐ Ecological, evolutionary & environmental sciences

For a reference copy of the document with all sections, see nature.com/documents/nr-reporting-summary-flat.pdf

Life sciences study design

All studies must disclose on these points even when the disclosure is negative.

Sample size	Sample size chosen based on POWER analysis of existing data
Data exclusions	No data exclusions were made.
Replication	Data from two independent trails were groups as indicated in the study for animal experiments. Samples were pooled from mulitple mice for RNA sequencing based studies to eliminate inter-animal variation.
Randomization	Animals were randomized into experimental groups based on baseline body weight measurements such that the mean body weight and body fat % between groups were not statistically different
Blinding	Blinding was done for histological, metabolic, micro-CT and RNA sequencing studies e.g., samples provided to cores in a blinded fashion but assessement of animal weights and body mass was not blinded due to fact experimenter had to remove mice from cages.

Reporting for specific materials, systems and methods

We require information from authors about some types of materials, experimental systems and methods used in many studies. Here, indicate whether each material, system or method listed is relevant to your study. If you are not sure if a list item applies to your research, read the appropriate section before selecting a response.

Materials & experimental systems

n/a	Involved in the study	
☐	☒	Antibodies
☒	☐	Eukaryotic cell lines
☒	☐	Palaeontology and archaeology
☐	☒	Animals and other organisms
☒	☐	Human research participants
☒	☐	Clinical data
☒	☐	Dual use research of concern

Methods

n/a	Involved in the study	
☒	☐	ChIP-seq
☐	☒	Flow cytometry
☒	☐	MRI-based neuroimaging

Antibodies

Antibodies used	CD11b (BD; 553309, 1:200), anti-CD34 (BioLegend; 119304, 1:200), anti-CD45 (BD; 553078, 1:200), CD16/32 Fc block (BD; 553142, 1:40), FITC anti-Ter119 antibody (Tonbo; 35-5921, 1:100), FITC anti-CD31 antibody (BD; 553372, 1:50), FITC anti-CD45 antibody (BD; 553078, 1:100), biotinylated anti-LEPR (R&D; BAF497, 1:33), donkey-anti-goat Alexa 647 (Invitrogen; A21447, 1:40), Tyrosine Hydroxylase (EMD Millipore; AB152 1:500) and Leptin (Novus Biologicals; MAB498, 1:4000).
Validation	Data on specificity and cross-reactivity was provided by the manufacturer and/or antibody clones were chosen based on previously published data.

Animals and other organisms

Policy information about studies involving animals; ARRIVE guidelines recommended for reporting animal research

Laboratory animals	3-month-old, male C57BL/6 mice were used in the study
Wild animals	Study did not involve wild animals
Field-collected samples	Study did not involve samples collected in the field
Ethics oversight	All animal studies were approved by the Institutional Animal Care and Use Committee of Scripps Florida

Note that full information on the approval of the study protocol must also be provided in the manuscript.

Flow Cytometry

Plots

Confirm that:

☒ The axis labels state the marker and fluorochrome used (e.g. CD4-FITC).

☒ The axis scales are clearly visible. Include numbers along axes only for bottom left plot of group (a 'group' is an analysis of identical markers).

☒ All plots are contour plots with outliers or pseudocolor plots.

☒ A numerical value for number of cells or percentage (with statistics) is provided.

Methodology

Sample preparation	Marrow from femurs and tibia was digested for 30 min with Liberase DL and DNAseI, red blood cells were lysed using the ACK lysis buffer, and then cells were immuno-depleted using biotinylated anti-CD11b, CD34, and CD45 antibodies and streptavidin coated magnetic beads. Remaining cells were stained with antibodies against Ter119, CD31, CD45 and LEPR and Lin-LEPR+ fraction obtained by FACS.
Instrument	BD FACS Aria
Software	FloJo software was used for post-acquisition analysis.
Cell population abundance	The abundance of the Lin-LEPR+ marrow population is ~0.17%, and purity was checked using functional assays including CFU-F activity and hematopoietic colony-forming activity
Gating strategy	FSC-A vs. SSC-A was used to eliminate debris, FSC-W vs. FSC-H was used to eliminate doublets/multiplets , and SSC-A vs. PI-A was used to eliminate dead cells. Lineage markers vs. LEPR was used to identify the Lin-LEPR+ fraction.

☒ Tick this box to confirm that a figure exemplifying the gating strategy is provided in the Supplementary Information.
